# Impact of Low Filter Resistances on Subjective and Physiological Responses to Filtering Facepiece Respirators 

**DOI:** 10.1371/journal.pone.0084901

**Published:** 2013-12-27

**Authors:** Raymond J. Roberge, Jung-Hyun Kim, Jeffrey B. Powell, Ronald E. Shaffer, Caroline M. Ylitalo, John M. Sebastian

**Affiliations:** 1 Technology Research Branch, National Personal Protective Technology Laboratory, National Institute for Occupational Safety and Health, Centers for Disease Control and Prevention, Pittsburgh, Pennsylvania, United States of America; 2 Personal Safety Division, 3M Company, St. Paul, Minnesota, United States of America; University of California, Merced, United States of America

## Abstract

Ten subjects underwent treadmill exercise at 5.6 km/h over one hour while wearing each of three identical appearing, cup-shaped, prototype filtering facepiece respirators that differed only in their filter resistances (3 mm, 6 mm, and 9 mm H_2_O pressure drop). There were no statistically significant differences between filtering facepiece respirators with respect to impact on physiological parameters (i.e., heart rate, respiratory rate, oxygen saturation, transcutaneous carbon dioxide levels, tympanic membrane temperature), pulmonary function variables (i.e., tidal volume, respiratory rate, volume of carbon dioxide production, oxygen consumption, or ventilation), and subjective ratings (i.e., exertion, thermal comfort, inspiratory effort, expiratory effort and overall breathing comfort). The nominal filter resistances of the prototype filtering facepiece respirators correspond to airflow resistances ranging from 2.1 - 6.6 mm H_2_O/L/s which are less than, or minimally equivalent to, previously reported values for the normal threshold for detection of inspiratory breathing resistance (6 - 7.6 mm H_2_O/L/sec). Therefore, filtering facepiece respirators with filter resistances at, or below, this level may not impact the wearer differently physiologically or subjectively from those with filter resistances only slightly above this threshold at low-moderate work rates over one hour.

## Introduction

Disposable filtering facepiece respirators (FFRs) are the most frequently used respirators in U.S. private industry and healthcare, and the N95 class of FFR (N95 FFR) is the single most selected version [1]. FFRs are classified as negative pressure respirators because inhalation against the resistance of the filter media creates pressure within the FFR deadspace (V_D_) that is negative with respect to ambient air pressure. U.S. Occupational Safety and Health Administration (OSHA)-mandated respiratory protection programs require the use of National Institute for Occupational Safety and Health (NIOSH)-certified FFRs. NIOSH certification limits for FFR inspiratory and expiratory filter resistance are 35 and 25 mm H_2_O pressure, respectively (tested at a constant airflow rate of 85 L∙min^-1^) [2]. These values are considerably less than the 60 - 140 mm H_2_O pressure that has been identified previously as being “noticeable but well tolerated” during the hardest work experienced by a worker wearing a half facepiece, elastomeric, negative pressure, air-purifying respirator [3]. Other comparable regulatory design requirements exist elsewhere (e.g., European Standard EN:149 indicates inhalation resistance limits of 7.1 and 24.4 mm H_2_O pressure, respectively, at 30 and 95 liters-per-minute of continuous airflow and exhalation resistance limits of 30.5 mm H_2_O pressure at 160 liters-per-minute of airflow). Many current models of N95 FFRs have demonstrated filter resistances of <25 mm H_2_O pressure during non-human testing at low-moderate work rates [4–6]. Jones [7] reported that, for subjects at rest and during 5 minute periods of light, moderate and high work rates, in-line pressure transducer measurements of FFR filter resistance were proportional to the work rate and ranged from 0-20 mm H_2_O pressure. The impact of filter resistance on airway pressures was investigated by Lee and Wang [8] who used a modified rhinomanometry method to demonstrate average nasal inspiratory and expiratory pressure increases of 0.32 Pa/cm^3^/sec (32 mm H_2_O/L/s) airflow when wearing an N95 FFR, an approximate doubling of baseline (no N95 FFR) nasal airway resistance [9]. Despite these relatively low resistances reported, complaints of breathing difficulty with N95 FFRs are nonetheless commonly expressed by a sizeable proportion of users in the healthcare industry [10–12]. The greater the resistance, the more energy expenditure is required for ventilation and the greater the perception of discomfort that can lead to decreased compliance with wear and, thus, compromise protection. All of this begs the question of the lowest feasible filter resistance that can be designed into N95 FFRs to maximize discernible (subjective) breathing comfort and minimize (objective) physiological strain while still maintaining adequate filtration and fit characteristics. The current study, part of NIOSH’s Project B.R.E.A.T.H.E. (Better Respiratory Equipment using Advanced Technologies for Healthcare Employees) [13], was carried out by the National Personal Protective Technology Laboratory (NPPTL) of NIOSH to investigate the subjective and physiological responses to wearing N95 FFRs with filter resistances lower than most standard N95 FFRs currently on the market. This data could be important for respirator manufacturers, standards development organizations, researchers, respiratory protection program managers and end users.

## Materials and Methods

Ten healthy, non-smoking subjects (7 men, 3 women) participated in this study, the majority of whom (8/10) were experienced N95 FFR users. Demographic mean values (standard deviations) were: age 24.5 years (3.8), height 179.0 cm (11.0), weight 75.3 kg (12.4), and body mass index 23.4 kg/m^-2^ (2.9). On the day of testing, subjects underwent a screening history and physical examination by a licensed physician. Subjects were dressed in athletic shorts or pants, tee shirts and athletic shoes during exercise testing. The study was approved by the NIOSH Human Subjects Review Board, and all subjects provided oral and written informed consent.

Identical appearing, cup-shaped, prototype FFR samples from 3M Company (St. Paul, MN, US) were supplied to NPPTL in nominal filter resistances of 3mm, 6mm and 9mm H_2_O pressure drop at 85 L/min of constant airflow (hereafter referred to as prototype respirators [PR] 3, 6, and 9, respectively) achieved through modifications of the filter media. Prior to study trials, pressure drop (i.e., pressure differential of airflow across the respirator filter) of the PRs was verified at NPPTL with the TSI 8130 automated filter tester (TSI, Shoreview, MN, USA) that is utilized for NIOSH respirator certification testing. Results indicated pressure drops of 3.6 mm, 6.5 mm and 9.3 mm H_2_O pressure for PR 3, PR6, and PR9, respectively, at 85 L/min constant airflow.. Subjects underwent standard OSHA respirator quantitative fit testing for each of the PRs with the TSI Portacount® Pro Respirator Fit Tester Model 8038 that counts the number of airborne particles in order to determine the ratio of ambient particles to within-respirator particles. The standard OSHA respirator quantitative fit test includes seven measured repetitive exercises of one minute duration each (i.e., normal breathing, deep breathing, head turn from side to side, bending head up and down, talking, bending over, normal breathing) and one 15 second exercise (grimace). Passing a fit test required a fit factor of ≥100, equivalent to a passing score on an OSHA quantitative respirator fit test and indicating that ≤1 in 100 particles was entering the FFR wearer’s breathing zone [14]. 

Subjects were instrumented with various sensors for continuous physiological monitoring during the exercise trials. The respiratory rate (RR) was monitored with the Zephyr Bioharness™ (Zephyr Technology Corporation, Annapolis, MD, US), an elasticized chest strap utilizing an proprietary embedded capacitive sensor to evaluate chest expansion and contraction [15,16]. The heart rate (HR), oxygen saturation (SpO_2_) and transcutaneous carbon dioxide (tcPCO_2_) were continuously monitored with the Tosca 500™ (Radiometer, Copenhagen, DM), an earlobe-mounted combination pulse oximeter and heated, Severinghaus-type CO_2_ sensor [17,18]. Tympanic membrane (ear) temperatures (T_tymp_) were measured immediately pre-and-post exercise trials with a Welch/Allyn Pro 4000 infrared tympanic thermometer (Braun GmbH, Kronberg, FRG). PR deadspace temperature (TV_D_) and relative humidity (RHV_D_) were continuously monitored with the iButton (Maxim, San Jose, CA, US), a small (16 mm x 6 mm) wireless sensor that was adhesively attached to the inner surface of the PRs midway between the respirator center and the edge of the right upper quadrant of the PR [19,20].

The study was carried out in a temperature and humidity controlled exercise physiology laboratory with mean (standard deviation) ambient conditions of: temperature 20.9 °C (1.3), relative humidity 23.5% (5.6), and barometric pressure 29.0 mm Hg (0.17). PRs were weighed pre-and-post testing on a calibrated Accu 6201 digital scale (Fisher Scientific, Pittsburgh, PA, US) to determine moisture retention. Immediately prior to each exercise study, measurement of some baseline pulmonary variables (i.e., tidal volume [V_T_], exhalation minute ventilation [V_E_], volume of carbon dioxide production [VCO_2_], volume of oxygen utilization [VO_2_]) was carried out utilizing a noseclip and tight-fitting mouthpiece connected to a Vmax Spectra respiratory analysis system (SensorMedics, Yorba Linda, CA, US) over a 5-minute period at sedentary (seated) activity. Subjects then donned a PR (PRs 3 and 9 were randomized; PR6 was not randomized because it was received from the manufacturer at a later date) according to the manufacturer’s instruction, performed positive and negative pressure user seal checks to assess the seal of the PR to the face [14], and walked on a treadmill at a low-moderate work rate (5.6 km/h) for 1 hour. Immediately upon completion of the exercise, and without interruption, the subject removed his/her PR and continued walking at the same rate on the treadmill for an additional 5 minutes while repeating measurements of the same pulmonary variables to approximate the impact, if any, of PR wear on pulmonary parameters. During the study trials, subjective measurements were taken immediately prior to the exercise period and then every 20 minutes using visual analog numerical scales for exertion (Borg Perceived Exertion Scale [a 15-grade scale ranging from “no exertion at all” to “maximal exertion]) [21], thermal comfort (Frank Comfort Scale [a 10-point scale ranging from “the coldest you have ever been” to “the hottest you have ever been”]) [22], and three 7-point respiratory scales for perceived inspiratory effort and perceived expiratory effort (ranging from “not noticeable” to “intolerable”) and overall breathing discomfort (ranging from “no discomfort” to “intolerable discomfort”) [23]. There was a minimum respite of 30 minutes between any consecutive exercise tests, during which subjects were seated and allowed to drink bottled water or a sports drink *ad lib*. 

### Statistical Analysis

The dependent variables in continuous measurements (SpO_2_, tcPCO_2_, HR, and RR) were summarized as means and standard deviations of 1 minute averages taken at 20 minute intervals throughout each trial (0, 20, 40, and 60 min), consistent with subjective measurement variables. A two-way, repeated measures ANOVA (Respirator × Time) with the Greenhouse-Geisser correction for assumption of sphericity was carried out to determine the main effect of each PR on physiological and subjective responses. For a significant main effect detected, a post-hoc pair-wise comparison with the least significant difference (LSD) adjustment was carried out. The non-continuous measurements in pulmonary data (pre- and post-trial) were also analyzed in the same procedure described above. A statistical significance was accepted when p<0.05 and all analyses were performed using a statistical software package (SPSS v18, IBM, Somers, NY).

## Results

All subjects passed each individual component of the respirator quantitative fit test for each of the PRs. There were no significant differences between the three PRs with respect to the physiological variables HR (F=0.37, p=0.63), RR (F=1.97, p=0.18), SpO_2_ (F=2.59, p=0.12), tcPCO_2_ (F=1.11, p=0.34), T_tymp_ (F=0.97, p=0.39) and pulmonary variables VO_2_ (F=0.78, p=0.47), VCO_2_ (F=0.47, p=0.63), V_T_ (F=0.89, p=0.42), and V_E_ (F=0.45, p=0.64) (Table 1). 

**Table 1:** Mean values (standard deviations) of physiological and pulmonary function variables measured when wearing prototype respirators.

**Trial / Time**	**0 Min¶**	**20 Min**	**40 Min**	**60 Min**
**SpO_2_ ( %)**				
PR3 (3mm H_2_O)	98.4 (0.8)	98.4 (0.6)	98.3 (0.4)	98.2 (0.5)
PR6 (6mm H_2_O)	98.5 (0.5)	98.6 (0.5)	98.5 (0.5)	98.4 (0.5)
PR9 (9mm H_2_O)	98.5 (0.7)	98.1 (1.2)	98.2 (0.7)	98.1 (0.6)
tcPCO_2_ (mm Mercury)*				
PR3 (3mm H_2_O)	41.3 (2.8)	43.1 (2.9)	42.4 (2.8)	41.8 (3.3)
PR6 (6mm H_2_O)	42.1 (2.4)	43.7 (3.7)	42.5 (4.2)	41.7 (4.1)
PR9 (9mm H_2_O)	40.6 (2.0)	42.0 (3.2)	41.6 (2.8)	41.4 (2.7)
HR (beats/min)*				
PR3 (3mm H_2_O)	83.0 (11.3)	102.1 (8.1)	104.8 (8.3)	104.9 (7.9)
PR6 (6mm H_2_O)	86.6 (11.8)	98.7 (9.4)	101.1 (10.2)	101.1 (9.8)
PR9 (9mm H_2_O)	78.9 (15.0)	100.4 (9.5)	103.1 (9.3)	106.3 (8.3)
RR (breaths/min)*				
PR3 (3mm H_2_O)	16.9 (6.0)	26.7 (5.9)	27.9 (5.4)	27.2 (6.1)
PR6 (6mm H_2_O)	18.8 (4.5)	29.0 (10.8)	28.7 (10.5)	28.0 (10.1)
PR9 (9mm H_2_O)	17.4 (5.6)	26.0 (5.5)	26.5 (5.1)	26.1 (6.8)
VO_2_ (mL/kg/min)*				
PR3 (3mm H_2_O)	4. (0.9)	-	-	17.9 (2.6)
PR6 (6mm H_2_O)	4.5 (0.6)	-	-	17.4 (3.9)
PR9 (9mm H_2_O)	4.8 (1.4)	-	-	17.8 (2.7)
VCO_2_ (mL/kg/min)*				
PR3 (3mm H_2_O)	3.5 (0.6)	-	-	13.9 (1.9)
PR6 (6mm H_2_O)	3.7 (0.7)	-	-	13.6 (2.6)
PR9 (9mm H_2_O)	3.9 (0.3)	-	-	14.1 (1.5)
V_E_ (L)*				
PR3 (3mm H_2_O)	10.4 (2.4)	-	-	31.8 (7.6)
PR6 (6mm H_2_O)	11.3 (2.0)	-	-	30.6 (7.0)
PR9 (9mm H_2_O)	11.4 (3.3)	-	-	32.1 (7.3)
V_T_ (L)*				
PR3 (3mm H_2_O)	0.7 (0.2)	-	-	1.2 (0.3)
PR6 (6mm H_2_O)	0.8 (0.2)	-	-	1.3 (0.3)
PR9 (9mm H_2_O)	0.8 (0.3)	-	-	1.3 (0.4)
T_Tymp_ (°C)*				
PR3 (3mm H_2_O)	36.5 (0.3)	-	-	36.9 (0.4)
PR6 (6mm H_2_O)	36.5 (0.3)	-	-	36.7 (0.2)
PR9 (9mm H_2_O)	36.6 (0.2)	-	-	36.8 (0.4)

SpO_2_ (pulse oximetry-derived oxygen saturation; tcPCO_2_ (partial pressure of transcutaneous carbon dioxide); HR (heart rate); RR (respiratory rate); VO_2_ (volume of oxygen utilization); VCO_2_ (volume of carbon dioxide production); V_E_ (minute ventilation); V_T_ (tidal volume); T_Tymp_ (tympanic temperature).

^*^ significant time effect.

^¶^ indicates the point at which the PR was donned

There were no significant differences between the three PRs in TV_D_ (F=0.05, p=0.94) and RHV_D_ (F=0.001, p=0.99) (Table 2), and mean moisture retention was ≤0.1 gram over one hour. No significant differences were noted between the three PRs in subjective scores for exertion (F=1.33, p=0.28), thermal comfort (F=1.08, p=0.33), inspiratory effort (F=0.008, p=0.96), expiratory effort (F=0.98, p=0.36), and overall breathing comfort (F=4.17, p=0.06) (Table 3). 

**Table 2:** Prototype respirator deadspace mean (standard deviation) temperature and relative humidity levels over one hour.

**Trial**	**TV_D_ (°C)**	**RHV_D_ (%)**
PR3 (3mm H_2_O)	32.3 (0.8)	83.5 (7.9)
PR6 (6mm H_2_O)	32.4 (0.6)	86.2 (5.6)
PR9 (9mm H_2_O)	32.4 (0.9)	86.3 (5.8)

**Table 3:** Mean (standard deviation) subjective scores reported while wearing prototype respirators over one hour.

**Trial / Time**	**0 Min**	**20 Min**	**40 Min**	**60 Min**
**Exertion (6: No exertion at all – 20: Maximal exertion)Ω**				
PR3 (3mm H_2_O)	7.0 (1.2)	9.0 (1.2)	10.1 (1.7)	10.2 (1.5)
PR6 (6mm H_2_O)	6.4 (0.5)	9.0 (1.3)	9.7 (1.5)	9.9 (1.7)
PR9 (9mm H_2_O)	7.3 (1.9)	9.1 (1.7)	10.0 (1.9)	10.4 (1.4)
Thermal Comfort (0: The coldest – 5: Neither hot nor cold – 10: The hottest)*§				
PR3 (3mm H_2_O)	4.3 (1.1)	5.1 (1.3)	5.7 (1.2)	5.8 (1.1)
PR6 (6mm H_2_O)	4.7 (0.5)	5.6 (1.0)	6.1 (0.7)	6.1 (0.7)
PR9 (9mm H_2_O)	4.6 (0.8)	5.5 (0.7)	5.9 (0.9)	6.2 (0.9)
Inspiratory Effort (1: Not noticeable – 7: Intolerable)*¶				
PR3 (3mm H_2_O)	1.6 (1.6)	2.2 (1.4)	2.3 (1.4)	2.5 (1.5)
PR6 (6mm H_2_O)	1.5 (1.3)	2.1 (1.2)	2.5 (1.2)	2.5 (1.2)
PR9 (9mm H_2_O)	1.4 (0.7)	2.1 (0.9)	2.4 (1.0)	2.5 (0.8)
Expiratory Effort (1: Not noticeable – 7: Intolerable)*¶				
PR3 (3mm H_2_O)	1.3 (0.5)	1.8 (0.6)	1.8 (0.6)	2.0 (0.9)
PR6 (6mm H_2_O)	1.3 (0.5)	2.0 (0.7)	2.3 (0.8)	2.3 (0.8)
PR9 (9mm H_2_O)	1.4 (1.0)	2.0 (0.9)	2.4 (1.2)	2.4 (1.2)
Overall Breathing Comfort (1: No discomfort – 7: Intolerable discomfort)*¶				
PR3 (3mm H_2_O)	1.2 (0.4)	1.4 (0.5)	1.6 (0.7)	2.0 (0.9)
PR6 (6mm H_2_O)	1.2 (0.4)	1.4 (0.5)	1.7 (0.8)	1.9 (0.7)
PR9 (9mm H_2_O)	1.4 (0.7)	1.8 (0.8)	2.1 (1.1)	2.4 (1.2)

ΩBorg Scale of Perceived Exertion (21)

^§^ Frank Scale of Perceived Comfort (22)

^¶^ From Antunano et al (23)

^*^ significant time effect

Time had a significant effect on tcPCO_2_ (F=3.57, p=0.02), inspiratory effort (F= 15.65, p=0.001), expiratory effort (F= 13.96, p=0.002), overall breathing comfort (F=9.47, p=<0.005), T_tymp_ (F=11.43, p=0.008), and all other physiological (HR [F= 30.19, p<0.01], RR [F=58.14, p<0.01]) and pulmonary function variables (VO_2_ [F=245.97, p<0.01], VCO_2_ [F=387.46, p<0.01] V_T_ [F=83.62, p<0.01], and V_E_ [F=125.51, p<0.01], save for SpO_2_ (F=1.39, p=0.27), TV_D_ (F=0.05, p=0.94), and RHV_D_ (F=0.01, p=0.99). There was also a significant time effect on all subjective scores; exertion (F=48.75, p<0.01), thermal comfort (F=32.59, p<0.01), inspiratory effort (F=15.65, p<0.05), expiratory effort (F=13.96, p<0.05), and overall breathing comfort (F=9.47, p<0.05).

## Discussion

### Physiological Responses

This study was undertaken to evaluate the role of one parameter, filter resistance, on physiological and subjective responses to wearing the three PRs. The study data demonstrate that, over one hour at a low-moderate work rate (5.6 km/h) consistent with that experienced by healthcare workers, three identical cup-shaped PRs, differing only in their low filter resistances (i.e., 3 mm, 6 mm, and 9 mm H_2_O pressure), did not vary significantly with regard to physiological and subjective impacts on the user. The lack of a significant difference in HR responses to the PRs (p=0.63) is supported by prior respirator research [7,17,24] showing that HR responses to various types of respiratory protective devices (i.e., FFRs, elastomeric negative pressure respirators, powered air-purifying respirators), at low and moderate work rates, were not statistically different from controls. Also, the non-significant differences in RRs in the current study (p=0.18) mirror previous findings between controls and subjects wearing various respiratory protective devices with higher resistances than the PRs (i.e., FFRs, full-facepiece negative pressure elastomeric respirators, self-rescuer escape respirators) at low-moderate work rates [17,23,25]. It is recognized that workload intensity has a greater physiological impact on HR and RR than the magnitude of inspiratory resistance [23]. The similarity in the mean values of the measured cardiopulmonary variables for all three PRs, despite their different filter resistances, suggests that the respirators had little impact on these variables (Table 1). SpO_2_ levels did not differ between PRs (p=0.12), as in other studies reporting normal SpO_2_ levels (≥95%) when wearing FFRs similar to the PRs in the present study [17,18,26]. Normal SpO_2_ levels are maintained, despite reported lower mixed inhalation/exhalation V_D_ O_2_ levels (16.6%) of FFRs at low work rates [17], because they correspond to an arterial O_2_ (PaCO_2_) level of 75 mm Hg [27] that is associated with 95% SaO_2_ on the oxygen-hemoglobin dissociation curve. As in the current study, there is a tendency towards an initial slight increase in baseline tcPCO_2_ levels of healthy individuals wearing respiratory protective devices (Table 1) that is due to the rebreathing of elevated V_D_ CO_2_ (e.g., 1.5% - 3.0% ) [17,18,28] and is compensable at V_D_ CO_2_ levels of up to 2% over short-term periods [29]. Significant elevations of tcPCO_2_ are related to respirator V_D_ volumes ≥350 mL [30]; the lesser V_D_ volume of the PRs (190 mL) is the likely reason for a lack of significant elevation in tcPCO_2_ levels among the PRs (p=0.34) and no significant elevation above normal levels in this study. 

Significantly elevated breathing resistance, above the normal airway resistance of 6 - 24 mm H_2_O/L/s, can have a negative impact on pulmonary function [31,32]. The principal physiological responses to added breathing resistance may include hypoventilation, reduced O_2_ consumption, increased respiratory work, a tendency for increased functional residual capacity, and increased CO_2_ retention [29]. The lack of significant differences of the PRs with regard to pulmonary parameters after one hour of exercise suggests that achievable decrements in filter resistance below 9 mm H_2_O pressure are unlikely to have significant beneficial impact on pulmonary function over 1 hour of FFR wear time at low-moderate work rates. 

### Subjective Perceptions

The absence of significant differences in the exertion scores between PRs (p=0.28) indicates that the subjects did not perceive differences in energy expenditure at the different filter resistances. Similar findings, using the same scale (Borg) as the present study, have been reported between controls and subjects wearing a cup-shaped FFR with a similar filter resistance to PR9 [33]. The related variables of thermal comfort and T_tymp_ were not significantly different between PRs (p=0.33 and 0.39, respectively) and reflect the fact that respiratory heat loss accounts for only a small portion (~10%) of the body’s heat transfer and that cup shaped FFRs cover <2% of the body’s skin surface involved in heat conduction, radiation and evaporation processes [33,34]. 

There were no significant differences in inspiratory and expiratory effort (p=0.94, 0.98, respectively) among PRs in the current study, with scores indicating that subjects perceived these breathing parameters as ranging from “not noticeable” to “noticeable but not difficult” [23]. The average filter resistances (pressure drop), as measured by the TSI 8130 automated filter tester at a constant flow rate of 85 L/min, were 3.6 mm, 6.5 mm, and 9.3 mm H_2_O pressure for PR3, PR6 and PR9, respectively. In order to estimate the mean pressure drop experienced by the PR wearers, the mean of the total exhaled V_E_ during one hour (31.5 L) (Table 1) was doubled to account for the inhalation component of respiration and resulted in a calculated mean flow rate of 63 L/min. The estimated pressure drop experienced by the subjects at a flow rate of 63 L/min would have been 2.6 mm, 4.8 mm, and 6.8 mm for PR3, PR6, and PR9, respectively. The greatest difference in the filter resistance that the subjects would have experienced would have been 4.2 mm H_2_O (i.e., between PR3 and PR9). Minimal inspiratory resistance pressure is considered to be 6 mm H_2_O/L/sec [35] and the threshold level of detection ranges from 6 - 7.6 mm H_2_O/L/sec [36–40]. Our calculated mean flow rate of 63 L/min corresponds to 6.3 mm H_2_O pressure, which is greater than the 4.2 mm H_2_O filter resistance differences among the tested PR, so that the added filter resistances of the PRs would be either undetectable or barely noticeable, as confirmed by the aforementioned subjective scores. It must be stated that these calculations are estimates only and may differ from actual real time measurements due to pulmonary-related variables (e.g., length of respiratory duty cycle, nasal versus oral breathing, etc.). Also, these calculations utilized an inspiratory:expiratory ratio of 1:1, rather than the usual 1:2 or 1:3 of human breathing such that our data may actually be underestimating the actual flow and pressure drop in inspiration. 

Expiratory effort scores were lower than inspiratory effort scores (Table 3), as previously reported in another study [32], and consistent with a detection threshold for expiratory resistance that is slightly higher than that for inspiratory resistance detection [36], as well as the fact that, at low-moderate work rates and low breathing resistances, expiration is a passive process. Overall breathing comfort scores were not significantly different (p=0.06) between PRs and indicated that the subjects’ perception of breathing comfort ranged from “no discomfort” to “slight discomfort”. This is not unexpected, given reports of no breathing discomfort in studies evaluating subjects wearing respiratory protective devices with breathing resistances many times greater (i.e., 40 - 100 mm H_2_O/L∙sec^-1^) than that of the PRs [23,25], although the test subjects in those studies were not representative of healthcare workers. Nonetheless, overall breathing comfort scores in the current study approached statistical significance and it must be appreciated that thresholds of breathing comfort are quite variable among individuals, so that subject factors other than breathing resistance (e.g., psychological, physiological and acclimatization inputs) may have been operant [23,41]. Further research involving PRs with filter resistances below 9 mm H_2_O pressure, with a larger number of subjects, may be needed to discern minor differences in subjective responses.

### Moisture Retention

The minimal moisture retention (≤ 0.1 gm) of the PRs, over one hour, is consistent with that noted in other FFR studies [5,17,18,42] and is a reflection of the breathability of modern respirators due to their thinness and porosity, and the fact that most FFR filters are made of polypropylene, a highly hydrophobic material. The passing of all fit tests by subjects in the current study relates, in significant measure, to the fact that the template for the PRs is the commercially-available 3M 1860 N95 FFR that has previously been shown to be associated with a high passage rate (>80%) on fit testing [10]. Time had a statistically significant effect on most study parameters and is indicative of the fact that continued FFR use imposes an increasing impact upon the user. 

### Limitations

Limitations of the current study include the relatively small number (n=10) of subjects tested. However, the majority of subjects (8/10) were experienced FFR users whose familiarity provided a higher level of insight into FFR-related issues and additional reliability to the data collected. The study subjects were relatively young, not overweight and experienced respirator users who may not be completely representative of respirator users in general, especially in light of the increasing prevalence of overweight and obesity in the U.S. population. Thus, the findings of this study may not be applicable to all respirator users. Although we were unable to collect measurements of pulmonary variables data while the subjects were wearing the PRs, prior investigations using impedance plethysmography have demonstrated that RR, V_T_ and V_E_ of controls are not significantly different from those wearing an N95 FFR at a work rate similar to the current study over one hour [17,18]. Also, while measurement of pulmonary variables using a noseclip may not be totally comparable to wearing a PR without a noseclip, some restriction of nasal airflow occurs due to respirator impingement on the nasal alae [43] and, as in the present study, the pliable nose bars of the PRs that are pinched at the nasal bridge area to conform to the nasal contour. This study evaluated only cup-shaped FFR, so that the data may not be applicable to other styles of FFR (e.g., flat fold, duck bill, etc.). Future studies could include testing other styles of FFR, as well as investigating respirator users who have experienced breathing difficulty with FFRs in the past. Our study was conducted at a low-moderate work rate, so that our data may not be applicable to higher work rates and associated increased airway resistance. Arterial blood gases are a more accurate indicator of ventilation performance (i.e., CO_2_ level) than tcPCO_2_, but the latter avoids painful needle punctures and provides continuous data that has been shown to correlate reasonably well with PaCO_2_ [44,45]. 

## Conclusion

Cup-shaped FFR PRs with filter resistances of 3 mm, 6 mm and 9 mm H_2_O pressure (measured at a constant airflow rate of 85 L/min), do not have significantly different impacts on physiological and subjective responses when worn for one hour at a low-moderate work rate (5.6 km/h). The level of detection of airway inspiratory resistance is 6 - 7.6 mm H_2_O/L/sec [36–40], so that cup-shaped FFRs with filter resistances in this range might not be perceived as significantly more comfortable than standard low-resistance FFRs with minimally higher filter resistance; however, given the small sample size of the current study (n=10), additional testing is warranted. Although many users of modern, low-resistance FFRs continue to complain of breathing difficulty [10–12], this may be related to numerous other inputs (e.g., warmth, anxiety/claustrophobia, alteration of breathing pattern from nasal to oronasal, etc.) such that efforts to decrease cup-shaped FFR filter resistance below 9 mm (measured at a constant airflow rate of 85 L/min) may prove of little benefit in terms of FFR breathing comfort and tolerance at low-moderate work rates. 
